# Using the Technology Acceptance Model to Explore Adolescents’ Perspectives on Combining Technologies for Physical Activity Promotion Within an Intervention: Usability Study

**DOI:** 10.2196/15552

**Published:** 2020-03-06

**Authors:** Mark Drehlich, Michael Naraine, Katie Rowe, Samuel K Lai, Jo Salmon, Helen Brown, Harriet Koorts, Susie Macfarlane, Nicola D Ridgers

**Affiliations:** 1 Institute for Physical Activity and Nutrition Deakin University Burwood Australia; 2 Deakin Business School Deakin University Burwood Australia; 3 Learning Futures Deakin University Burwood Australia

**Keywords:** fitness trackers, social media, physical activity, youth

## Abstract

**Background:**

Wearable activity trackers and social media have been identified as having the potential to increase physical activity among adolescents, yet little is known about the perceived ease of use and perceived usefulness of the technology by adolescents.

**Objective:**

The aim of this study was to use the technology acceptance model to explore adolescents’ acceptance of wearable activity trackers used in combination with social media within a physical activity intervention.

**Methods:**

The Raising Awareness of Physical Activity study was a 12-week physical activity intervention that combined a wearable activity tracker (Fitbit Flex) with supporting digital materials that were delivered using social media (Facebook). A total of 124 adolescents aged 13 to 14 years randomized to the intervention group (9 schools) participated in focus groups immediately post intervention. Focus groups explored adolescents’ perspectives of the intervention and were analyzed using pen profiles using a coding framework based on the technology acceptance model.

**Results:**

Adolescents reported that Fitbit Flex was useful as it motivated them to be active and provided feedback about their physical activity levels. However, adolescents typically reported that Fitbit Flex required effort to use, which negatively impacted on their perceived ease of use. Similarly, Facebook was considered to be a useful platform for delivering intervention content. However, adolescents generally noted preferences for using alternative social media websites, which may have impacted on negative perceptions concerning Facebook’s ease of use. Perceptions of technological risks included damage to or loss of the device, integrity of data, and challenges with both Fitbit and Facebook being compatible with daily life.

**Conclusions:**

Wearable activity trackers and social media have the potential to impact adolescents’ physical activity levels. The findings from this study suggest that although the adolescents recognized the potential usefulness of the wearable activity trackers and the social media platform, the effort required to use these technologies, as well as the issues concerning risks and compatibility, may have influenced overall engagement and technology acceptance. As wearable activity trackers and social media platforms can change rapidly, future research is needed to examine the factors that may influence the acceptance of specific forms of technology by using the technology acceptance model.

**Trial Registration:**

Australian and New Zealand Clinical Trials Registry ACTRN12616000899448; https://www.anzctr.org.au/Trial/Registration/TrialReview.aspx?id=370716

## Introduction

### Background

Engaging in regular physical activity is critical for adolescent health. Higher physical activity levels support weight management, musculoskeletal development, fitness, cardiovascular health [1], and mental health through enhanced self-concept and reduced anxiety and depression [2]. For adolescents (aged 12-17 years), the Australian Government recommends 60 min of at least moderate-intensity physical activity daily [3], yet only 18% of adolescents meet these guidelines [4]. With physical inactivity being recognized as a global pandemic [5] and an estimated 80% of the global adolescent population classified as inactive [6], strategies are needed to increase levels of physical activity.

To date, many physical activity interventions have either been reported to be ineffective within inactive populations or unscalable because of cultural, geographic, social, or economic contexts [7]. It is further posited that adolescents perceive many interventions negatively as individuals tend not to self-select into such interventions; rather such interventions are imposed on them by others [8]. Opportunities exist to utilize novel approaches to encourage adolescents to participate in physical activity. Given the increasing popularity of wearable activity trackers, a potential strategy for increasing youth physical activity levels is to examine how such devices might be used to encourage physical activity among adolescents. Wearable activity trackers are electronic devices that use sensors to track movement and collect biometric data [9] and enable constant self-monitoring through the provision of feedback via a visual display and/or accompanying app [10]. As such, these devices allow individuals to have an enhanced awareness of self, and these devices have the potential to generate internal motivation for physical activity.

Past research indicates that interventions that use wearable activity tracking devices may be acceptable to adolescent populations; therefore, wearable activity trackers have the potential to increase adolescents’ levels of physical activity [9,11,12]. More recently, studies have explored how wearable activity trackers and forms of social media can be combined in interventions targeting adolescent inactivity [13-15]. Social media (eg, Facebook and Instagram) has emerged as a popular communication medium, offering expedited connectivity and engagement [16-18]; therefore, it can be used to provide additional support for physical activity. However, little is known about how adolescents engage with such technology within a physical activity intervention [9], and few studies have explored an individual’s engagement within intervention components using theoretical models that may help to provide insights into such use [12]. For example, a potentially important feature of wearable activity trackers is the ability to share data to and receive peer support via social media [19]. Such engagement and support from others may lead to an increase in motivation and reinforcement to participate in physical activity [20]. The strategic use of social media to engage adolescents could result in stronger bonds that lead to increased feelings of relatedness and increased engagement in physical activity [21], and may provide a source of motivation to participate in physical activity [13,22]. Existing physical activity studies in adolescents and young people have found that frequently engaging with social media is associated with increased physical activity [14,19], although the challenge is identifying the strategies to engage adolescents, particularly as some research suggests that social media engagement is often passive [13,15]. However, little research has examined the individual’s perceptions and experiences of using social media platforms, such as Facebook, when combined with physical activity interventions using wearable activity trackers. Such information could help to inform strategies to achieve optimum intervention impact and engagement among adolescents.

### Technology Acceptance Model

A framework that enables researchers to examine technology use is the technology acceptance model [23]. This model provides a framework for evaluating how different factors may influence an individual’s use and acceptance of specific forms of technology, such as wearable activity trackers or social media [23]. In addition, more recent advances in the technology acceptance model have included the perceived risks associated with using specific forms of technology and the degree of compatibility that such technology has with an individual’s values and needs [24,25]. Overall, the focus of the model is not on whether the technology results in increased levels of physical activity but on how the different technology used in a physical activity intervention is accepted by a target group. Whether a target group is willing to accept and use the specific forms of technology relied upon in a physical activity intervention is important to understand, given it is unlikely that an intervention will lead to increased levels of physical activity among a target group if that group does not accept or is unwilling to use the chosen forms of technology.

To date, few studies have used the technology acceptance model to examine factors that may influence the acceptance of specific forms of technology, such as wearable activity trackers and social media, when combined within a physical activity intervention. In studies that have used the technology acceptance model, the focus has been on adults rather than adolescents. For example, Lunney et al [26] found that perceived usefulness significantly influenced adults’ acceptance of wearable activity trackers, whereas perceived ease of use was a direct determinant of their behavior (use of activity trackers). Similarly, Chuah et al [27] found that perceived usefulness can assist in determining adults’ attitudes toward wearable activity trackers, but it did not predict their adoption intention (intention to use the device). Opportunities exist to extend the understanding of technology acceptance from adults to adolescents.

### Objective

The aim of this study was to use the technology acceptance model to explore adolescents’ acceptance of wearable activity trackers and social media when used in combination within a physical activity intervention.

## Methods

### Study Design and Participants

**Figure 1:**
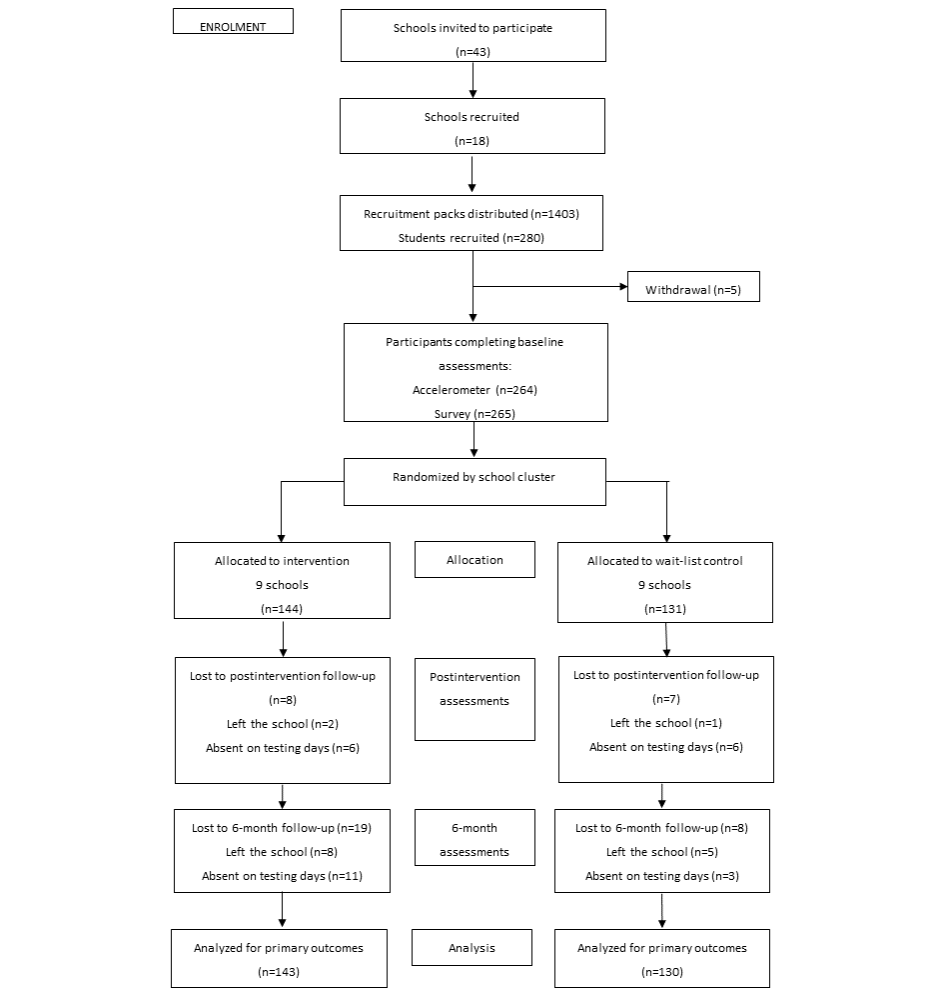
Flow of participants throughout the study. This study focused on the data collected at post-intervention.

The design and methods of the study have been reported in detail elsewhere [28]. In brief, the Raising Awareness of Physical Activity (RAW-PA) study was a 12-week multi-component study that combined a wearable activity tracker and digital behavior change resources delivered via social media, which aimed to increase inactive adolescents’ physical activity levels. Schools located in areas that had a Socio-Economic Indexes for Areas [29] in the lowest 50% and were within approximately 60 km of Deakin University’s Burwood Campus were eligible to participate. A total of 18 schools (42% response rate) were recruited (Figure 1). Participants were adolescents in Year 8 (aged 13 to 14 years) who self-reported that they did not engage in regular physical activity/sport, did not meet current physical activity guidelines, had not previously owned or used a wearable activity tracker, had (or were willing to create) a Facebook account, and had access to the internet outside of school (age: mean 13.8 years, SD 0.4 years; 142/275, 51.6% female). Ethics approval for this study was obtained from the Deakin University Human Research Ethics Committee and the Victorian Department of Education and Training. Informed consent to participate in the study was obtained from all schools and parents, with written assent provided by adolescents. The study is registered with the Australian and New Zealand Clinical Trials Registry (ACTRN12616000899448).

### Intervention

Adolescents (n=144) attending schools that were randomized to the intervention group (n=9) received a wrist-worn Fitbit Flex and an accompanying app, as well as access to the interactive weekly individual or team “missions” and behavior change resources via a private, researcher-moderated Facebook group [28]. Facebook was chosen as the form of social media as, at the time of the study, it was the most popular social media platform [30]. The aim of the Facebook group was to provide adolescents with a platform to ask questions, interact with other participants, and engage with posted content that related to the weekly missions. Alerts for new content were also sent to the adolescents through email and/or text messages (approximately 2-3 times per week) [28]. The intervention components and structure were developed using participatory research principles, and the combination of technologies aimed to target low-cost forms of physical activity (eg, walking) and guide adolescents through the behavior change process in a way that was accessible, flexible, and interactive [28].

At the start of the intervention, the research team provided initial assistance in setting up Fitbit Flex, which included creating a Fitbit account for each participant and providing information on how to sync and charge the device and how to use the app to view data. No other information was provided at this time about the use of Fitbit Flex. Adolescents were informed that new content would be posted regularly to the Facebook group, though no guidance was provided about the frequency with which to access content.

### Theoretical Framework

**Figure 2:**
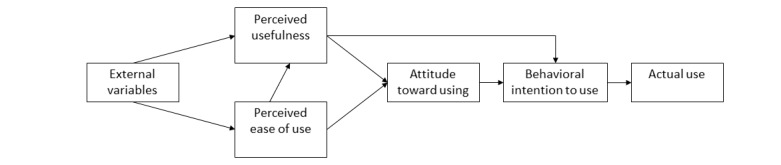
Technology acceptance model.

This study utilized the technology acceptance model [23] to examine adolescents’ perspectives on combining wearable activity trackers and social media within a physical activity intervention (Figure 2). The technology acceptance model identifies 2 variables that are key to technology acceptance and use: (1) perceived ease of use (is using the technology free from effort?) and (2) perceived usefulness (will the use of the technology enhance performance?). According to the technology acceptance model, perceived ease of use and perceived usefulness, either alone or in combination, predict behavioral intention (intention to use technology), which in turn predicts subsequent behavior (actual use of technology) [23]. Specifically, this model is being used to examine whether the different technologies used in a physical activity intervention can be easily used by the target group and whether such technology offers valued benefits to the target group [23].

### Measures

To address the research questions in this study, only data collected from the student focus groups were used. At the end of the 12-week intervention period, all adolescents attending intervention schools were invited to participate in focus groups that explored their thoughts and perspectives on RAW-PA and the different components within the intervention. In total, 124 students (63 males and 61 females; 124/144, 86.1% of the intervention group) participated in 15 focus groups that took place at each school (n=9). Focus groups contained both males and females and ranged from 6 to 13 participants (average of 8 participants). This enabled the adolescents to provide unique insights into their experiences of RAW-PA and their acceptance of the technologies used in this study, thus enabling us to respect their expert knowledge and lived experience [31]. The focus groups followed a semistructured format that was designed to identify potential enablers and barriers to using different components of RAW-PA, which included a discussion of the key forms of technology used—Fitbit Flex and Facebook. Example questions included “What did you like/not like about the Fitbit and accompanying app?,” “What did you like/not like about the Facebook group?,” “Did you experience any issues using aspects of the program (eg, Fitbit or Facebook)?,” and “Did anything help you to use the different features (eg, Fitbit or Facebook)?.” Focus groups (mean duration 26 min) were digitally recorded and transcribed verbatim, producing 256 pages (Times New Roman, Font 12) of data for analysis.

### Data Analyses

#### Focus Group Data

Qualitative data were analyzed using pen profiles, an increasingly used technique for presenting findings to researchers with qualitative and quantitative backgrounds [31-33]. Pen profiles present key themes identified during data analysis through the combination of verbatim quotes taken directly from the transcripts to provide context with frequency data [31,34]. The numbers reported against each theme indicate the number of times the theme was cited in focus groups, as individuals in focus groups were not identified. Data were initially analyzed using a deductive process in which the technology acceptance model [23] was used to develop a coding framework and inform the coding of the concepts of perceived usefulness, perceived ease of use, perceived risk, and compatibility in relation to the key technological components of this intervention: (1) the wearable activity tracking device, Fitbit Flex, and (2) the social media platform, Facebook. Perceived ease of use was defined as “the degree to which a person believes that using a technology will be free from effort” [35]. Perceived usefulness was defined as “the extent to which a person believes that using particular technology will enhance their performance” [35]. An inductive coding process was then used to identify the key themes that emerged from the data [31,36].

As recommended by Burnard [37], a researcher (MD) who was independent of the project delivery team initially read and analyzed the transcripts. Following the development of the pen profiles, the findings were then presented to 2 independent researchers with expertise in the use of technology for activity promotion and qualitative data analyses (KR and MN). Data from the pen profiles to the transcripts were cross-examined, which enabled alternative interpretations and data interrogation until an overall consensus was achieved. The pen profiles were then presented to 2 project delivery team members (NR and SL) who further critically challenged the interpretation of the data. Credibility and transferability were demonstrated through the triangular consensus procedure and verbatim transcription of collected data [33,34].

## Results

### Findings

Data concerning the perceived ease of use and perceived usefulness of Fitbit Flex and Facebook are presented initially. As the adolescents often discussed Fitbit Flex and the accompanying app interchangeably, it should be noted that results concerning Fitbit include both the device and the app unless otherwise stated. The findings that relate to the perceived risk and compatibility of these technologies are then presented in the last pen profile.

### Fitbit: Perceived Ease of Use

**Figure 3:**
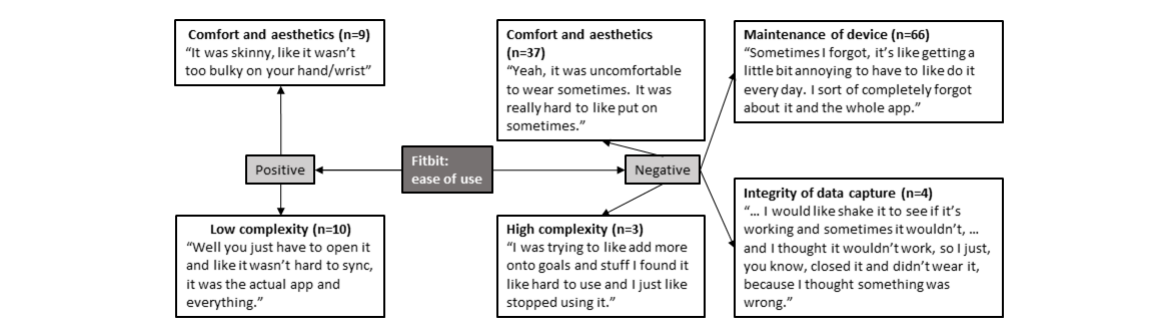
Perceived ease of use of Fitbit in adolescents. Note: n=number of times theme was mentioned by adolescents.

With respect to the perceived ease of use of the device (Figure 3), although some focus group participants indicated that the device was easy to use, many adolescents reported that it was not free from effort. For example, they explained that the device was hard to put on; they needed to take it off for some activities (ie, swimming and playing a sport); they often forgot to wear, charge, and sync the device; or they had technical issues while charging or syncing the device. Several Fitbit functions were also perceived negatively, with the primary concerns being maintenance requirements, including the life of the battery (need to charge it frequently), and the need to sync the data regularly, all of which required a concerted effort to address. In addition, the integrity of the data was challenged at times, with adolescents unsure as to how the device captured their data, whether it was capturing data at all times, and if the data were accurate. A few adolescents reported that they perceived the device negatively because Fitbit Flex did not have a screen display, which detracted from their ease of using the device to track their activity levels.

### Fitbit: Perceived Usefulness

**Figure 4:**
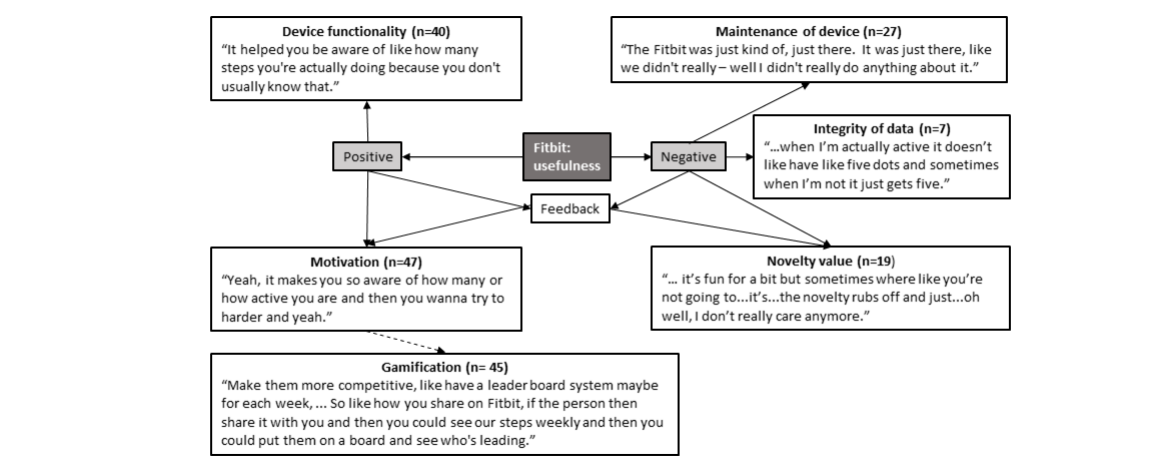
Fitbit’s perceived usefulness in adolescents. Note: n=number of times theme was mentioned by adolescents.

From the perspective of perceived usefulness, adolescents reported that Fitbit Flex’s specific functions and its ability to motivate them to participate in physical activity were positive aspects of the device (Figure 4). Other reported benefits included the device providing adolescents with a greater awareness of their physical activity patterns and a greater level of motivation to engage in physical activity in response to such knowledge. Participants also noted that Fitbit Flex was useful for setting goals and evaluating whether these had been achieved. These features were perceived as being useful to a number of adolescent participants. However, it was also frequently mentioned that Fitbit Flex may be more useful and motivating if additional gamification offerings were provided, such as the recognition of achievement via the device or app. In addition, some concerns were raised in relation to whether the data captured were meaningful and, therefore, useful. Specifically, concerns were raised regarding the potential for differences to exist between actual and recorded activity levels. This made the adolescents question the usefulness of the device for the purposes of monitoring activity levels. Moreover, the lack of a display also impacted perceived usefulness, especially as adolescents reported little engagement with the app to obtain information about their activity levels, meaning that feedback from the device was often limited or perceived to be insufficient for their needs. Adolescents also recognized that there was a diminishing return on using Fitbit Flex because of an initial novelty factor of wearing the device, which existed when they commenced the program but diminished over time, again impacting perceived usefulness.

### Facebook: Perceived Ease of Use

**Figure 5:**
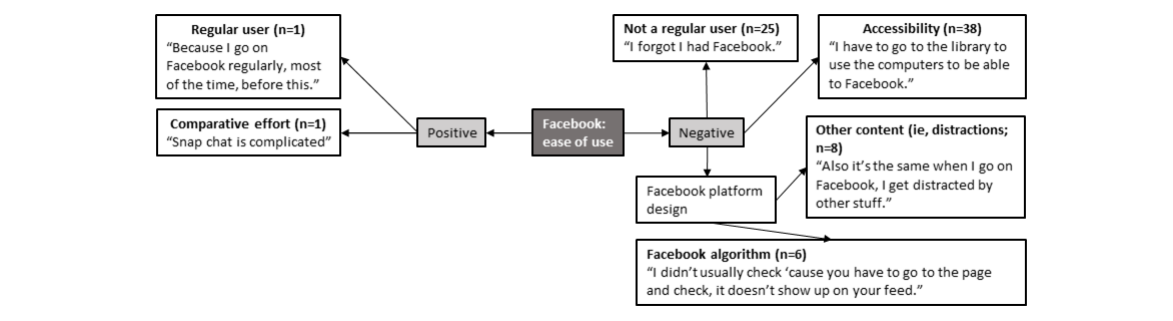
Perceived ease of use of Facebook in adolescents. Note: n=number of times theme was mentioned by adolescents.

Adolescents’ perceptions in relation to the perceived ease of use of Facebook within the intervention are summarized in Figure 5. Overall, negative perceptions regarding perceived ease of use were most commonly expressed. These reflections included that the design of and perceived effort to use Facebook diminished its ease of use, that the platform design comprised several independent elements that were not well connected, and that adolescents who were irregular users of Facebook had to intentionally log in to engage with RAW-PA content. Others identified that they did not want to use their mobile phone data allocation for accessing intervention content on Facebook; therefore, access became an issue. Adolescents also recognized that if they were not actively seeking to engage with RAW-PA content on Facebook (ie, intentionally looking for it), the intervention materials would not appear in their news feed because of the algorithms Facebook uses. Interestingly, several adolescents noted that they would get distracted by other content, which meant reductions in the frequency of the introduction of RAW-PA into the feed because of Facebook’s algorithm. Only 2 positive comments relating to the perceived ease of use of Facebook were reported, namely, previous exposure to the platform and the relative ease of use in comparison with other platforms.

### Facebook: Perceived Usefulness

**Figure 6:**
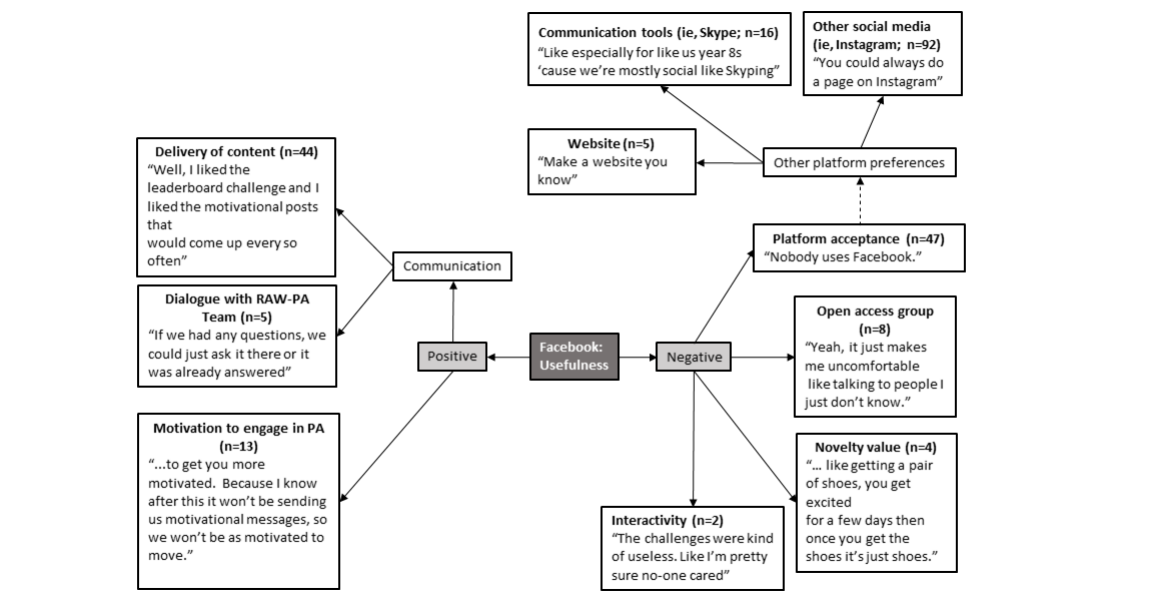
Perceived usefulness of Facebook in adolescents. PA: physical activity; RAW-PA: Raising Awareness of Physical Activity. Note: n=number of times theme was mentioned by adolescents; --- indicates an extension of a theme.

Adolescents’ perspectives on Facebook’s perceived usefulness are shown in Figure 6. Adolescents spoke positively about how useful Facebook was for delivering content in relation to the RAW-PA intervention. In particular, many adolescents stated that the RAW-PA messages provided through Facebook were useful in motivating them to engage in additional physical activity. Some adolescents considered Facebook a useful communication platform, albeit with the research team rather than their peers. The most commonly discussed limitation in relation to using Facebook was its lack of acceptance as a social media platform, thus decreasing its perceived usefulness. Adolescents highlighted their preference for having “private” groups that would be inaccessible to other members of the Facebook group to reduce the risk of their comments and messages being shared with the wider intervention group. Although Facebook had some initial value at the start of the program, as this novelty wore off, adolescents reported that their interest; use; and, ultimately, the usefulness of the platform diminished. Adolescents indicated a preference for alternative platforms, including stand-alone apps and websites, image and video platforms (ie, Snapchat and Instagram), or direct communication tools (ie, Skype).

### Perceived Risk and Compatibility

**Figure 7:**
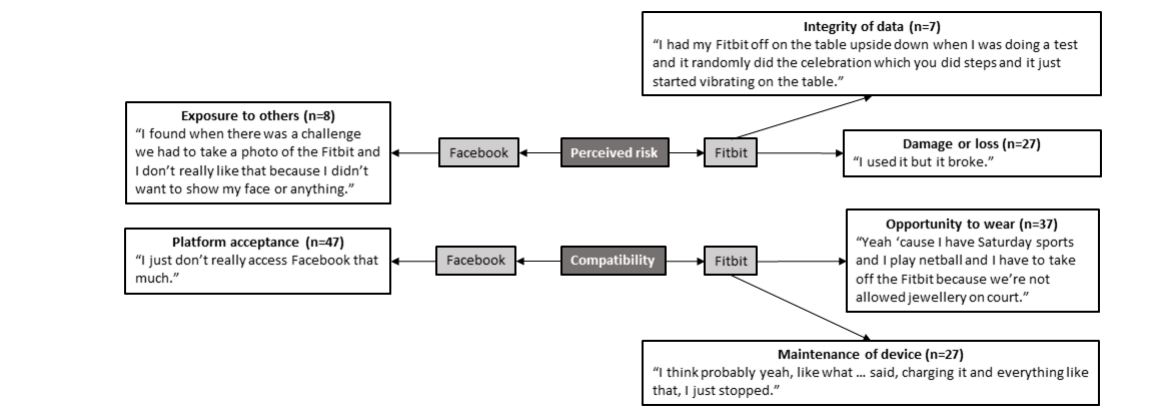
Facebook and Fitbit perceived risk and compatibility.

Several risks of using these technologies were perceived by the adolescents (Figure 7). When discussing Facebook, the commonly reported perceived risk was exposure to others within the intervention*.* Further related to Facebook, the lack of compatibility with the adolescents’ lifestyle was commonly alluded to. Facebook was considered a platform that adults used and was out of touch with the needs of adolescents. The risks surrounding the use of Fitbit Flex included the integrity of the data as well as the potential to damage or lose the device while using it. Adolescents also stated that the effort required to use the device (eg, regularly charging and syncing the device) was incompatible with their lifestyle.

## Discussion

### Principal Findings

The aim of this study was to utilize the technology acceptance model to explore adolescents’ acceptance of wearable activity trackers (Fitbit Flex) and social media (Facebook) when combined within a physical activity promotion intervention. Overall, adolescents generally reported that they perceived Fitbit Flex to be useful for tracking physical activity and motivating them to participate in physical activity, but perceptions concerning the perceived ease of use were often negative. Issues concerning the device’s ease of use, need for regular charging and syncing, and functionality were discussed as factors that required effort to either address or understand. Similarly, Facebook had more positive responses concerning its perceived usefulness, particularly as a vehicle to deliver motivating content in relation to Fitbit Flex, although it was no longer the preferred social media platform for adolescents in this study. However, perceived ease of use was low because of the design of the platform and the effort required to use it. Concerns were also raised about the compatibility of the technologies with current lifestyles and risks associated with using the technologies. In general, the findings suggest that the adolescents recognized the potential usefulness of the wearable activity trackers and social media platform in a physical activity intervention, but the effort required to use the technologies, as well as issues concerning risks and compatibility, may have led to lower technology acceptance.

### Comparison With Previous Work

To date, few studies have utilized the technology acceptance model [23] to examine the acceptance of technology within a physical activity intervention. In relation to wearable activity trackers, this study found that the functionality offered and the motivation generated by Fitbit Flex were important factors in the perceived usefulness of the device. Previous research has shown that the functionality of wearable activity trackers is important to the wearer, particularly in relation to self-tracking activity levels throughout the day [11,38]. Moreover, others have identified that wearable activity trackers can increase the wearer’s awareness and understanding of their own physical activity levels, which in turn provide motivation to engage in physical activity [14,20]. Indeed, Schafer et al [38] clearly identified that lack of motivation was a barrier to engagement and subsequent adoption, therefore support was required to continue engagement of adolescents.

The perceived usefulness of the device was potentially negatively impacted by diminished novelty over time. This is consistent with previous research, which has noted that novelty effects reduce the device’s perceived usefulness and therefore the probability that a wearer will continue to use the device after progressing past the intention to use it [9,20,38-40]. Interestingly, little research has examined whether personal preferences for specific wearable activity trackers may affect the perceptions of usefulness, with most studies providing one specific device for use. Future studies could consider providing different wearable activity tracker options to see if this helps sustain use over time. Of note, adolescents commented that incorporating specific digital game elements (ie, gamification) could benefit the perceived usefulness of wearable activity trackers, such as Fitbit Flex, within a physical activity intervention. Wider research [41,42] supports this finding, identifying positive outcomes because of gamified approaches and reductions in physical inactivity post intervention. Notably, gamified elements, such as competition and digital recognition for efforts, were incorporated into RAW-PA; although given the negative feedback provided concerning the usefulness of Facebook to deliver such elements, it is possible that this may have been missed by participants. As such, future studies should consider incorporating gamified elements [41] such as leaderboards, competitions, and tangible rewards into a physical activity intervention to enhance the usefulness of wearable activity trackers and engagement with the device. Although this has the potential to increase engagement and motivation [13,26], whether or not this will address concerns of novelty effects requires further investigation.

In this study, perceptions of Fitbit Flex’s ease of use were typically negative. For example, the device needed to be charged and synced regularly for it to operate and collect data, which adolescents indicated took more effort than warranted. It was commonly reported that adolescents forgot to wear the device after it had been removed, which is consistent with previous research [20,40]. Some concerns were raised about the wearability of the device, which also impacted its ease of use. Specifically, adolescents reported that the device was uncomfortable, it took effort to tolerate wearing it, and the device’s clasp was problematic. Rupp et al [43] noted similar issues with adolescents having difficulty while putting on the device, whereas others have highlighted how the comfort and design of the device can be a barrier to adolescents’ use of the technology [12,20,38]. In contrast, some adolescents perceived that the design of Fitbit Flex meant that it was considered to have good wearability. Interestingly, there was general agreement on the fact that Fitbit Flex was comparatively simple to use, and little instruction was required, which is consistent with previous studies [11,12]. Overall, these findings suggest that a wearable activity tracker’s perceived ease of use is a critical component of a technology-based intervention, and future studies should identify potential strategies to overcome perceived barriers to ease of use among adolescents.

In recent years, there has been an increase in the number of studies using Facebook to deliver physical activity interventions in different populations [13-15,19,21,44]. As is the case in this study, Facebook has often been chosen based on its popularity [21] and the opportunity it offers to provide information and social support to the user [13]. There was some indication that Facebook was perceived to be useful for receiving intervention content, communicating with the research team, and providing some motivation through social support. This is consistent with the findings of Pumper et al [13] who suggested that using Facebook in physical activity interventions directed at adolescents may be motivating and increase engagement, although it was noted that active rather than passive engagement of adolescents may be required to provide a source of extrinsic motivation [15]. Interestingly, research has suggested that Facebook provides motivation for engagement through individuals likening themselves to others (perceived role models and peers) and receiving gratification through the approval of others [21], as well as updates and messages using inspirational imagery, which promote higher levels of engagement [22]. However, adolescents need to engage with it for it to be effective, which was perceived to require effort.

Identifying the strategies to encourage social support and approval of others may be important for future interventions. Although Facebook was the dominant social media platform for adolescents and informed the intervention design at the time of study development, it was evident that it was no longer the preferred social media platform for adolescents during this study [12]. Furthermore, some adolescents did not like sharing information with others they did not know from other schools. Pumper et al [13] supported this, noting that although passive engagement (viewing content) was common among adolescents, active engagement (contributing content) was uncommon. Although this study used a private group, adolescents appeared to perceive contributing and participating in the group forum as a risk, which likely reduced their engagement. Divine et al [21] supported this, noting that through social comparison, Facebook has the potential to encourage or discourage engagement. The challenge for future physical activity interventions is to identify social media platforms that meet the needs of the users and therefore optimize their acceptance of these social media platforms. It may be that the interventions may need to be available on a number of different platforms, although this may be challenging given the ever-changing nature of social media. Future research should consider the dynamic nature of social media and implications for use during interventions. This further speaks to the need for communities to be organically developed and not forced into existence [13].

The perceived ease of use of Facebook in this group of adolescents was generally low. Of concern, some noted that the algorithms used by Facebook were perceived to be detrimental to engagement, as the intervention content was either lost among other content or did not appear in their news feed. Edney et al [22] recently identified that social media algorithms impacted news items in an adolescent’s feed, suggesting that participants would have to actively seek items as a part of the intervention. Interestingly, several adolescents in this study mentioned that Facebook’s accessibility also impacted its perceived ease of use, as they did not want to use their allocation of mobile phone data to access the platform; this finding is consistent with previous studies [12,45]. This meant that adolescents would have to seek internet access from alternative sources of data (eg, library, public Wi-Fi, and school computers) to access Facebook, which took additional effort.

The strengths of this study included the use of qualitative methods to explore adolescents’ thoughts and experiences in depth after engaging in a 12-week physical activity intervention and utilizing the technology acceptance model as a framework. However, there are several limitations that should be noted. First, although adolescents reported that they had not used a wearable activity tracker previously, it is unknown whether their expectations of the device may have impacted their subsequent experiences of using the device. Second, the lack of adolescents’ engagement with and willingness to share through social media, as well as the impact this would have on intervention implementation, was not anticipated. Third, although majority of the intervention students participated in focus groups, it is unknown as to whether their perspectives differed from those who did not participate. Fourth, data were collected at the end of the 12-week intervention. How the perceived usefulness and ease of use of Fitbit Flex and Facebook may have changed over time is also unknown. This information is valuable in developing future interventions and understanding the needs of adolescents in such interventions. Further research could consider assessing how adolescents’ attitudes toward such technology evolve throughout a study. Fifth, the qualitative data in each of the pen profiles represent the number of times the theme is mentioned and not the number of individuals who agree with the viewpoint. Although this is a common approach with pen profile analyses [33], it is possible that the data do not represent the views of all those who participated in the focus group and only represent the views of those who responded to the questions. However, it is possible that others may agree with certain points, and they may have felt that the point was made and chose not to reiterate. Finally, as not all participants were involved in the focus groups, we were unable to determine whether those participating in the focus groups had more favorable perspectives (for example) than those who did not participate.

### Conclusions

There is potential for both wearable activity trackers and social media to positively impact physical activity interventions among adolescents. However, this study highlighted the importance of perceived usefulness, perceived ease of use, perceived risk, and compatibility for understanding how adolescents engage with such technologies. Although both the wearable activity tracker and social media platform were considered useful, concerns about their ease of use, perceived risks associated with such use, and compatibility issues appeared to be critical and led to a low level of acceptance of and engagement with the technology. Technology advances rapidly, and interventions that use technology to engage with adolescents should continue to monitor and evaluate how technologies are used and accepted within physical activity interventions. The technology acceptance model can provide a useful framework to examine how technology is accepted among target groups, such as adolescents.

## Abbreviations

RAW-PA: Raising Awareness of Physical Activity
